# Offshore Oil Spill Detection Based on CNN, DBSCAN, and Hyperspectral Imaging

**DOI:** 10.3390/s24020411

**Published:** 2024-01-10

**Authors:** Ce Zhan, Kai Bai, Binrui Tu, Wanxing Zhang

**Affiliations:** 1 Hubei Key Laboratory of Drilling and Production Engineering for Oil and Gas, Yangtze University, Jingzhou 430023, China; 2021710596@yangtzeu.edu.cn (C.Z.); 2021710622@yangtzeu.edu.cn (B.T.); 2021710638@yangtzeu.edu.cn (W.Z.); 2 Xi’an Key Laboratory of Tight Oil (Shale Oil) Development, Xi’an Shiyou University, Xi’an 710065, China; 3 School of Computer Science, Yangtze University, Jingzhou 430023, China

**Keywords:** offshore oil spill, artificial neural network, hyperspectral image

## Abstract

Offshore oil spills have the potential to inflict substantial ecological damage, underscoring the critical importance of timely offshore oil spill detection and remediation. At present, offshore oil spill detection typically combines hyperspectral imaging with deep learning techniques. While these methodologies have made significant advancements, they prove inadequate in scenarios requiring real-time detection due to limited model detection speeds. To address this challenge, a method for detecting oil spill areas is introduced, combining convolutional neural networks (CNNs) with the DBSCAN clustering algorithm. This method aims to enhance the efficiency of oil spill area detection in real-time scenarios, providing a potential solution to the limitations posed by the intricate structures of existing models. The proposed method includes a pre-feature selection process applied to the spectral data, followed by pixel classification using a convolutional neural network (CNN) model. Subsequently, the DBSCAN algorithm is employed to segment oil spill areas from the classification results. To validate our proposed method, we simulate an offshore oil spill environment in the laboratory, utilizing a hyperspectral sensing device to collect data and create a dataset. We then compare our method with three other models—DRSNet, CNN-Visual Transformer, and GCN—conducting a comprehensive analysis to evaluate the advantages and limitations of each model.

## 1. Introduction

During the course of marine oil exploration, development, and transportation, un-foreseen accidents and spills can have profound repercussions. These incidents result in substantial harm to the marine environment. Simultaneously, the oil contains volatile organic compounds, and their evaporation contributes to atmospheric pollution. This destruction and pollution not only pose significant threats to marine ecosystems but also directly jeopardize human economic activities and health [1,2,3]. Upon the occurrence of an oil spill incident, the necessity arises to implement appropriate measures for its mitigation, thereby reducing its environmental repercussions. The timely and accurate detection of offshore oil spills assumes paramount importance in the domain of emergency response and the effective management of offshore oil spill incidents. Upon the occurrence of an oil spill incident, it is imperative to implement measures aimed at mitigating its adverse environmental effects. The timely and precise detection of marine oil pollution assumes utmost importance in the context of emergency response and the effective management of offshore oil spill events. Currently, offshore oil spill monitoring predominantly hinges upon data acquisition conducted by vessels, aircraft, and remote sensing satellites, subsequently followed by oil spill detection through data analysis algorithms [4]. Remote sensing exhibits a multitude of advantages, including a wide array of data sources and extensive monitoring capabilities. Nonetheless, it is susceptible to the influence of weather conditions and cloud cover, and it faces challenges in detecting smaller-scale oil spill areas. In contrast, close-range monitoring conducted by vessels or low-altitude aircraft is less susceptible to the interference of weather conditions and cloud cover [5]. Moreover, it demonstrates heightened sensitivity to small-scale oil spill areas. In the context of offshore oil spill response operations, vessels are typically equipped with hyperspectral sensing devices. Their primary mission is the precise detection and identification of small-scale oil spill areas, coupled with the requirement for the real-time monitoring of these regions. In such circumstances, algorithmic precision and performance take on increased significance [6]. In recent years, scholars have integrated hyperspectral data with machine learning and deep learning methods, resulting in a series of significant accomplishments [7,8,9,10,11,12]. Wang Dawei and his team introduced an improved deep learning model for oil spill detection called BO-DRNet. This model classifies pixels in hyperspectral images to identify oil spill areas, addressing issues such as insufficient feature extraction and fixed hyperparameters that are prevalent in traditional models [13]. Furthermore, they investigated the application of an improved semantic segmentation model called DRSNet based on support vector machines in marine oil spill monitoring. This study provides valuable methods for enhancing offshore emergency management capabilities [14]. JunFang Yang et al. introduced an oil spill detection model rooted in graph convolution architecture and spatial–spectral information. Through a comparative analysis with the GCN and CEGCN models, this approach demonstrated a notable enhancement in detection accuracy [15]. Seyd Teymoor Seydi et al. utilized a one-dimensional multiscale residual convolutional neural network to classify pixels based on the spectral features of oil spill region pixels, aiming to achieve the detection of oil spill areas [16]. Saeid Dehghani-Dehcheshmeh et al. combined CNN and Visual Transformer (ViT) methodologies to segment images into two categories: oil spill and background, effectively achieving oil spill detection [17]. The aforementioned methods have made significant advancements in the detection of oil spill areas in marine environments, underscoring the immense potential of hyperspectral imaging in this domain. However, these methods have relatively complex model structures. The complexity of these structures requires stronger computational power for model execution. In maritime oil spill response operations, aircraft or vessels equipped with hyperspectral sensors find it challenging to carry powerful computing systems, thus failing to meet the real-time detection demands. This challenge has prompted researchers to continue exploring more efficient algorithms and technologies to enhance the feasibility of hyperspectral imagery in detecting oil spills in marine areas. This necessitates the development of lighter-weight models or the adoption of more efficient analytical methods. Ongoing innovation in this research field will contribute to better meeting the real-world demands of real-time detection while continuing to leverage the crucial role of hyperspectral imagery in environmental monitoring and oil spill emergency response.

In this study, we simulated offshore oil spills in the laboratory and used a hyperspectral sensing device to capture images, validating our method. To address the issue of slow detection speeds resulting from overly complex models, this paper proposes an oil spill monitoring method based on convolutional neural networks (CNNs) and the DBSCAN clustering algorithm. The method includes a pre-feature selection step that eliminates spectral features with minimal contributions to the model. This enables the model to concentrate specifically on oil spill area detection. Leveraging the oil spill hyperspectral images that we collected, we selected three models—DRSNet, CNN-Visual Transformer, and GCN—for comparative analysis against the method proposed in this study. The method proposed in this paper features lower complexity than the other models, achieving a similar recognition accuracy and demonstrating a faster detection speed. Due to the lower model complexity and faster detection speed of the method proposed in this study, it meets the requirements of real-time detection scenarios.

## 2. Data Acquisition and Preprocessing

**Figure 1:**
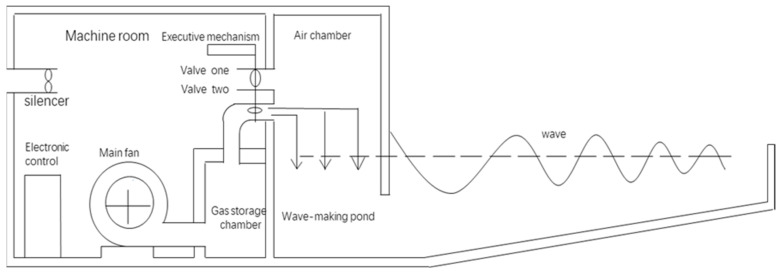
Wave pool.

In this study, data collection was carried out by simulating offshore oil spill scenarios and employing hyperspectral sensing equipment. The essential experimental apparatus included a wave pool (Figure 1), a hyperspectral camera, and a computer. The study categorized experiments into four groups based on the presence of oil and the existence of waves, collecting a total of 504 hyperspectral image samples. In our experiments, we were able to simulate different wave parameters by adjusting the main fan speed and controlling valves. Our experimental equipment is capable of measuring wave parameters, including wave height, wavelength, wave period, and wave speed. As a method for simulating marine environments, it has inherent limitations. The size of the water tank is relatively small compared to the real environment, making it challenging to simulate large-scale waves. Additionally, variations in water depth in the natural ocean may influence the propagation and shape of waves, whereas the experimental environment maintains a fixed water depth. Furthermore, the wind in the real environment affects waves, but due to experimental constraints, we could not simulate the effects of wind. Natural waves also exhibit nonlinear characteristics, making it difficult to simulate such complexity in experiments. Due to these factors, we opted to simulate smaller-scale regular waves in our experiments, with a wave height of 0.2 m, a wavelength of 2 m, a wave period of 2 s, and a wave speed of 1 m per second. These parameters closely resemble calm waves under light wind conditions.

We randomly selected a portion of hyperspectral images from each group to create the training dataset, while the remaining images were reserved for evaluating our constructed models. The dataset division is detailed in Table 1. Given the nature of hyperspectral images, it is essential to extract spectral data on a per-pixel basis. Consequently, from the hyperspectral images used to build the training dataset, we extracted 500 pixel-based spectral data samples from both the oil spill and non-oil spill regions, with corresponding annotations.

## 3. Theoretical Framework and Method Design

### 3.1. Method Design

**Figure 2:**
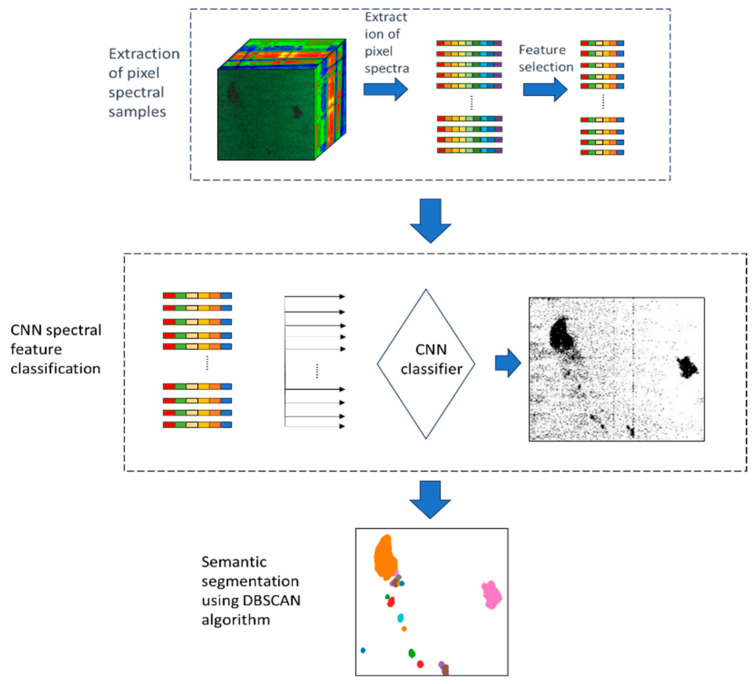
Steps of the oil spill area detection method.

For detecting oil spill areas from the collected hyperspectral images, our proposed oil spill area detection method primarily utilizes a convolutional neural network (CNN) classification model and the DBSCAN clustering algorithm. CNN is a widely used model for classification tasks, leveraging convolutional and pooling layers to effectively capture meaningful features of pixel spectra. The convolutional operations enable adaptability to features of different scales. The DBSCAN clustering algorithm is well suited for regions with significant density variations. It excels in adapting to differences in density across various areas in an image, requiring no predetermined number of clusters. This algorithm can identify clusters of arbitrary shapes and exhibits robustness to noise. By combining the advantages of CNN and the DBSCAN clustering algorithm, our method is better suited for segmenting hyperspectral images. In accordance with Figure 2, our devised method for oil spill area detection from the collected hyperspectral images encompasses three primary steps:

(1) The extraction of pixel spectra from hyperspectral images and feature selection to construct pixel spectral samples.
(2) The classification of pixel spectral samples using a convolutional neural network (CNN)-based model.
(3) The segmentation of oil spill area from the classification results using the DBSCAN algorithm.

In the detection process, the CNN model is responsible for pixel classification, while the DBSCAN clustering algorithm is tasked with segmenting the oil spill areas from the classification results. Due to the robustness of the DBSCAN algorithm to noise, it can effectively ignore noise pixels resulting from CNN classification errors. Therefore, there is no need for a highly accurate CNN classification model to precisely detect oil spill areas from hyperspectral images. Because a high-precision CNN model is not required, we can adopt a simpler structure. Additionally, the parallel processing nature of CNN enables the rapid processing of large-scale hyperspectral image data. Furthermore, DBSCAN is an unsupervised learning algorithm that does not require prior labels, making it suitable for real-time detection scenarios.

### 3.2. Spectral Feature Selection

**Figure 3:**
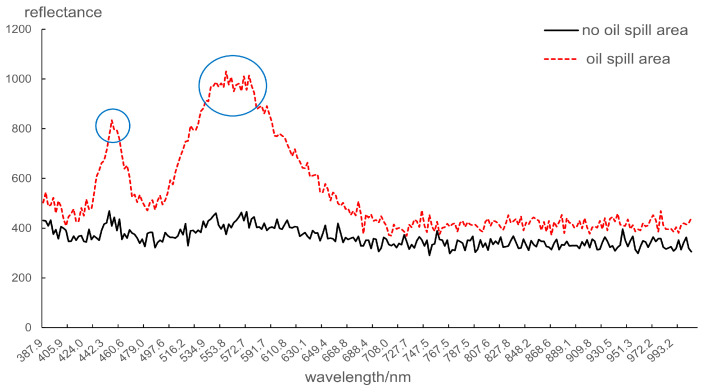
Spectral reflection intensity curve in oil spill and no oil spill area.

In the oil spill simulation experiment, we collected spectral reflectance data in the range of 400 to 1000 nm, comprising a total of 256 spectral features. Figure 3 illustrates two spectral reflectance intensity curves obtained during the experiment. The dashed line represents the spectral reflectance intensity curve for the non-oil spill area, while the solid line represents the reflectance intensity curve for the oil spill area. Upon observation, the spectral data exhibit a high dimensionality, with significant differences in spectral characteristics between the oil spill and non-oil spill areas apparent only in specific bands. As shown in Figure 3, there are two peaks in the spectrum of the oil spill area, one at 449.1 nm and the other in the range of 539.6 to 579.1 nm. Oil contains various compounds, mainly aromatic compounds, hydrocarbons, asphaltene, and some heavy oil. These compounds exhibit different spectral characteristics to water, so these two peaks may be attributed to the influence of these compounds present in oil and natural light. In practical machine learning applications, datasets typically comprise a multitude of features. Among these features, there may be many unimportant ones, which can easily lead to the following two shortcomings [18]:

(1) A large number of features can increase the model’s training time, affecting its efficiency.
(2) A large number of features can lead to the “curse of dimensionality”, increasing the model’s complexity and weakening its generalization capability.

Therefore, we need a method to reduce the dimensionality of the dataset, filtering out features that are not helpful for problem solving. This can enhance the efficiency and generalization ability of the model. The Random Forest (RF) algorithm is a commonly used feature selection method in feature engineering. It provides a method to measure feature importance [19,20], and the steps are described as follows:

(1) Calculate the baseline accuracy: Before constructing the Random Forest, start by calculating the baseline accuracy, which is the accuracy of the model when no features are considered.
(2) Rank a specific feature: For each feature, randomly shuffle its order, disrupting its relationship with the target variable.
(3) Recalculate accuracy: Train the model using the shuffled feature and calculate the new accuracy.
(4) Compute the decrease in accuracy: The decrease in accuracy is equal to the baseline accuracy minus the new accuracy obtained using the shuffled feature.
(5) Repeat steps 3 and 4: Repeat the process multiple times, permuting the same feature multiple times, and calculate the Mean Decrease Accuracy, which is the average decrease in accuracy.

Mean Decrease Accuracy (*MDA*) is a relative indicator used to assess feature importance, and it can be described using Equation (1):

(1) $$\begin{array}{c} {MDA}_{C}\left(j\right)=\frac{1}{T}\sum_{t}^{T}\left[\frac{1}{\left|D_{t}\right|}\left(\sum_{X_{i}\in D_{t}}I\left(P\left(X_{i}\right)==y_{i}\right)-\sum_{X_{i}^{j}\in D_{t}^{j}}I\left(P\left(X_{i}^{j}\right)==y_{i}\right)\right)\right] \end{array}$$

where *T*: the number of random trees constructed in Random Forest (RF).

$X_{i}$, $y_{i}$: $X_{i}$ represents the input samples, and $y_{i}$ represents the corresponding sample categories or labels.

$D_{t}$: sample set out of pocket for random tree *T*.

$D_{t}^{j}$: the sample set formed after exchanging the *j*-th feature.

$P(X_{i})$: the predicted outcome for sample $X_{i}$.

$I(P(X_{i})==y_{i})$: indicator function, which returns 1 if the predicted outcome is the same as the true category and 0 otherwise.

**Figure 4:**
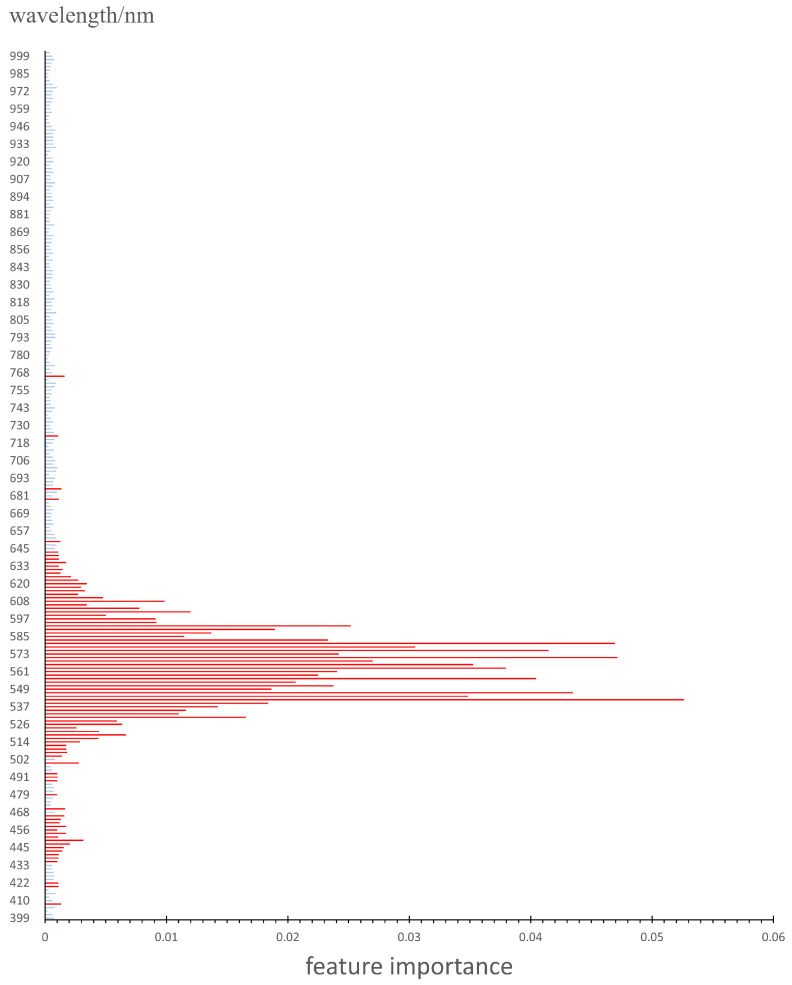
The importance of the 256 spectrum features.

Equation (1) reflects the degree to which the accuracy of the model’s classification decreases when random exchanges are made on the data in the *j*-th feature dimension. This calculation is based on the out-of-bag data for each tree. If the accuracy significantly decreases after random exchanges, it is considered that the corresponding feature is important. Therefore, Mean Decrease Accuracy (*MDA*) is regarded as a measure of feature importance. We extracted 500 samples of pixel spectra from oil spill areas and 500 samples from non-oil spill areas in the hyperspectral images. Following the steps of the Random Forest algorithm as mentioned above, we computed the importance of 256 wavelength features, and the results are shown in Figure 4. Based on the results of the Random Forest algorithm, we excluded features with importance less than 0.001. The features marked in red in Figure 4 represent the retained 88 features. These 88 wavelength features are used in the subsequent model construction process. The complexity of the model is mainly determined by the number of model parameters and the model structure. By reducing the number of input features from 256 to 88, the quantity of model parameters is directly reduced, leading to a decrease in model complexity.

### 3.3. CNN Classification Module

In the field of deep learning, a popular neural network architecture is the convolutional neural network (CNN), which is widely used for classification and image recognition tasks [21]. A CNN mainly consists of four key layers: an input layer, convolutional layer, pooling layer, and fully connected layer. The general process of a CNN is as follows:

(1) Data normalization: First, normalize the input data to accelerate model training and reduce the impact of noisy data on the model.
(2) Feature extraction through convolution: The convolutional layers employ convolutional kernels to extract relevant features from the input data.
(3) Pooling down-sampling: The pooling layer reduces the dimensions of the output data from the convolutional layers, preserving essential features while compressing the data.
(4) Classification through the fully connected layer: The primary role of the fully connected layer is classification. It aggregates, classifies, and adjusts network weights based on neuron feedback, ultimately generating the classification results.

**Figure 5:**
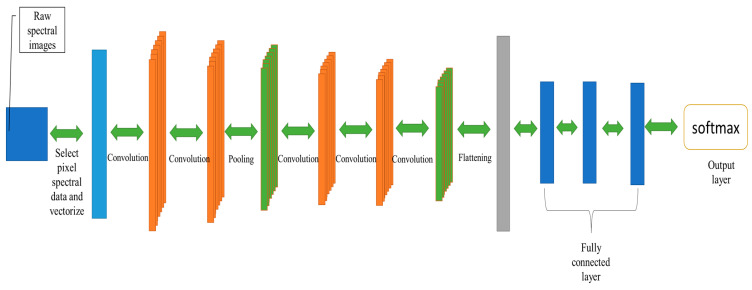
Structure of the CNN classifier.

In the proposed method of this research, the role of the CNN model is to classify pixels based on their spectral features. Compared to other classification models, the core strength of CNN lies in its convolutional layers, which can rapidly extract classification features. This advantage becomes particularly pronounced in scenarios with high-dimensional feature spaces where classification efficiency is crucial. After feature selection, there are now only 88 features remaining in the spectral data. These features contribute significantly to pixel spectral classification, so the input for the CNN model should consist of these 88 feature data points to form a feature vector. The CNN classifier structure is as shown in Figure 5. The model comprises one input layer, four convolutional layers, two pooling layers, and three fully connected layers. The convolutional layers employ 1 × 4 convolutional kernels, and the pooling layers have a stride of 2. Table 2 provides a detailed list of the parameters for each layer in the model.

**Figure 6:**
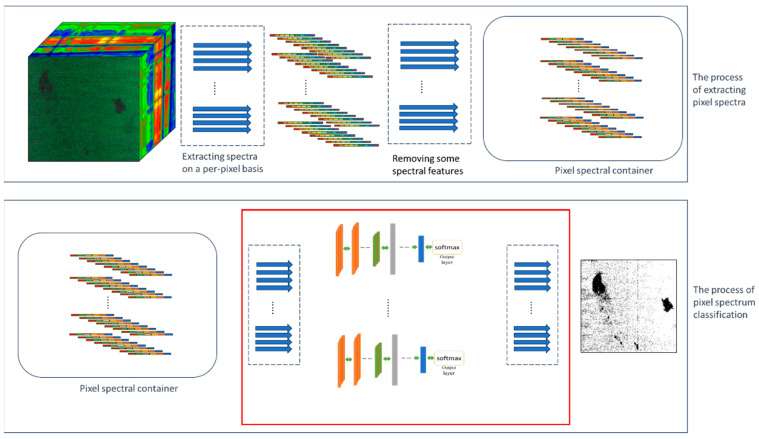
Process of pixel spectrum extraction and classification.

Upon the completion of the CNN pixel spectral classifier, the next step involves establishing a pixel classification module for hyperspectral image classification. Since the simultaneous classification of every pixel in hyperspectral images is required, it is essential to develop a parallel pixel spectral classification method. This module consists of two main processes: the first one is the extraction of pixel spectra, and the second is the classification of these pixel spectra. If these two processes are carried out sequentially, it will lead to a significant increase in time consumption and a failure to fully maximize computational resources. As each pixel’s classification is an independent process, the extraction and classification of pixel spectra can be conducted simultaneously, as depicted in Figure 6. The pixel spectral extraction process comprises two stages: spectral extraction and feature selection. Spectral samples, after undergoing feature selection, enter the pixel spectral container and await classification by the CNN classifier. The spectral extraction process and the classification process occur concurrently. Once all pixel classifications are completed, the classification results can be input into the semantic segmentation model to segment the oil spill areas.

### 3.4. Image Segmentation Based on the DBSCAN Algorithm

DBSCAN is a density-based spatial clustering algorithm that is robust to noise. This algorithm treats regions with sufficient density as distance centers and continually expands these regions. The algorithm is based on the fact that a cluster can be uniquely determined by any of its core objects. It utilizes the concept of density-based clustering, which requires that the number of objects contained within a certain region (eps) in the clustering space is not less than a given threshold (minPts). And it can discover clusters of arbitrary shapes in spatial data with noise. It connects adjacent regions with sufficiently high density, making it effective for clustering and handling outliers [22]. From an intuitive standpoint, this clustering algorithm can effectively identify dense regions within the sample points and offers the following advantages:

(1) Cluster analysis can be performed without the need to specify the number of clusters in advance.
(2) Cluster analysis can be applied to dense datasets of arbitrary shapes.
(3) Anomalous data points are unlikely to exert a substantial impact on the clustering results and can be discerned during the clustering process.

**Figure 7:**
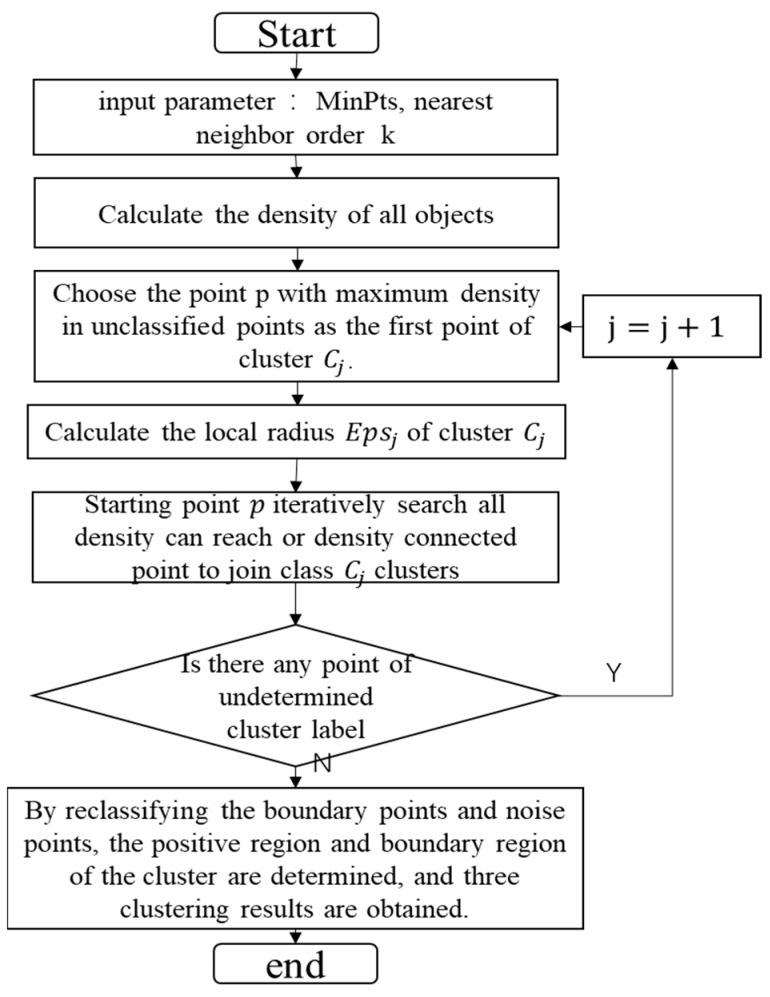
The process of DBSCAN.

In hyperspectral images, the shape and quantity of oil spill areas are both unknown. Additionally, due to imaging conditions and potential errors in the CNN classifier, noise is prevalent. In such a scenario, the application of the DBSCAN algorithm in oil spill detection holds significant value. It helps mitigate the impact of both inherent noise and CNN classification errors. The DBSCAN clustering algorithm process is depicted in Figure 7. This algorithm commences from an unvisited data point and identifies all adjacent points within a distance less than or equal to eps. If the count of neighboring points is greater than or equal to minPts, the current point initiates a cluster with its neighbors and marks the starting point as visited. Subsequently, all unvisited points within the cluster undergo the same process, resulting in the expansion of the cluster. If a cluster expands to a sufficient size where all its points are marked as visited, the same steps will be applied to other unvisited points. In this study, due to the presence of inherent noise and classification errors of the CNN classifier, there are some pixels within the samples for clustering that do not belong to the oil spill region. However, their distribution in the image is relatively sparse. Therefore, selecting a smaller eps and a larger minPts effectively mitigates the impact of these noise points.

## 4. Experimental Results and Evaluation

This study introduces a novel method for oil spill area detection. We designed two sets of experimental processes to assess its performance. First, we compared it with other oil spill detection methods by establishing multiple oil spill detection approaches on the same dataset and comparing these models using predefined evaluation criteria. Second, we retrained the model with a reduced number of training samples to validate its robustness.

### 4.1. Evaluation Methods

We applied the method proposed in this study to a simulated oil spill hyperspectral image dataset and compared it with three other models: DRSNet, CNN-Visual Transformer, and GCN. The evaluation of these methods was based on five metrics: Pixel Accuracy (*PA*), Oil Spill Class Pixel Accuracy (*OSCPA*), Non-Oil Spill Class Pixel Accuracy (*NOSCPA*), Mean Pixel Accuracy (*MPA*), and their mean detection time (*MT*). The metrics *PA*, *OSCPA*, *NOSCPA*, and *MPA* were calculated based on the confusion matrix, as shown in Table 3. The confusion matrix contains four types of data [23]:

(1) *TP*: Pixels classified as oil spill areas and are indeed oil spill area pixels are referred to as True Positives.
(2) *FP*: Pixels classified as oil spill areas but, in reality, are non-oil spill area pixels are referred to as False Positives.
(3) *TN*: Pixels classified as non-oil spill areas and are indeed non-oil spill area pixels are referred to as True Negatives.
(4) *FN*: Pixels predicted as non-oil spill area pixels but are actually oil spill area pixels are referred to as False Negatives.

Here are the definitions for *PA*, *OSCPA*, *NOSCPA*, and *MPA* based on the confusion matrix, as given in Equations (2)–(5):

(2) $$PA=\frac{TP+TN}{FP+FN+TP+TN}$$

(3) $$OSCPA=\frac{TP}{TP+FP}$$

(4) $$NOSCPA=\frac{TN}{TN+FN}$$

(5) $$MPA=\frac{1}{k}\times \left(\frac{TP}{TP+FP}+\frac{TN}{TN+FN}\right)$$

where *k* is the number of classes.

To assess the detection speed of the method proposed in this study, we used the test samples that were divided in the first part, comprising a total of 70 hyperspectral image samples. We applied this method and three comparative models to detect oil spill areas in these hyperspectral images. According to Equation (6), we calculated the average time required for each of the four models to detect a single hyperspectral image. This evaluation was conducted to assess the recognition speed of the method.

(6) $$MT=\frac{1}{p}\times \sum_{i=1}^{p}{Time}_{i}$$

where *p* represents the number of samples in a hyperspectral image, and *Time* represents the time consumed for sample detection.

### 4.2. Results and Comparative Analysis

**Figure 8:**
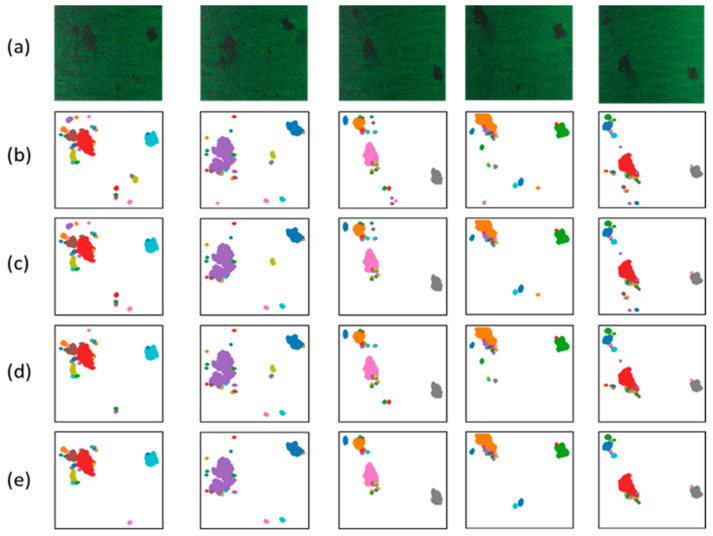
Partial sample detection results for the four methods. (**a**) The original hyper spectrum images. (**b**) Results of CNN-DBSCAN. (**c**) Results of DRSNet. (**d**) Results of CNN-Visual Transformer. (**e**) Results of GCN.

In Figure 8, we present the detection results of the method proposed in this study and three comparative methods on partial test samples. Group ‘a’ represents the original samples, while groups ‘b’, ‘c’, ‘d’, and ‘e’ correspond to the results of the model proposed in this study, DRSNet, CNN-Visual Transformer, and GCN, respectively. In Figure 8, it can be observed that the method proposed in this study, along with the other three methods, exhibits similar oil spill detection capabilities. All four methods can effectively detect the main oil spill areas. Compared to the original samples, CNN-DBSCAN appears to be more sensitive to oil spill areas. It accurately identifies even smaller oil spill areas but may also misclassify some non-oil spill areas as oil spill areas. In contrast, the other three methods exhibit fewer misclassifications of oil spill areas, but they perform slightly worse in recognizing small-scale oil spill areas. Among them, the GCN model shows a more noticeable weakness, with more small-scale oil spill areas being missed. Considering the recognition performance for both oil spill and non-oil spill areas, the four methods demonstrate similar capabilities in detecting oil spill areas. However, the model proposed in this study excels at detecting smaller oil spill areas.

In the previous sections, we presented the performance of the four methods on a subset of samples and provided a qualitative analysis of the comparison between the method proposed in this paper and the other three methods. To quantitatively demonstrate the performance of the four methods, we use the five metrics mentioned earlier to showcase the test results: Pixel Accuracy (*PA*), Oil Spill Class Pixel Accuracy (*OSCPA*), Non-Oil Spill Class Pixel Accuracy (*NOSCPA*), Mean Pixel Accuracy (*MPA*), and their mean detection time (*MT*). First, each of the four methods is applied to detect the 70 samples in the test set. Subsequently, the confusion matrix parameters *TP*, *FP*, *TN*, *FN*, and the time required to complete the detection for these 70 samples are recorded and analyzed. Then, the performance metrics, including *PA*, *OSCPA*, *NOSCPA*, *MPA*, and *MT*, are calculated for each of the four methods using Equations (2)–(6). The results are presented in Table 4.

The Pixel Accuracy (PA) for all four methods is consistently above 90%. Specifically, DRSNet and CNN-Visual Transformer exhibit slightly higher PA than the method proposed in this study, while GCN’s Pixel Accuracy is on par with that of the proposed method. For oil spill area detection, the objective is to detect as many oil spill areas as possible. Therefore, Class Pixel Accuracy (CPA) is a more important metric for evaluating accuracy. The method proposed in this study has an OSCPA of 89.21%, slightly lower than the other three methods. However, NOSCPA is significantly higher than that of the other three methods, and MPA is comparable to that of the comparative methods. In addition to comparing class-specific pixel accuracy, the Class Average Pixel Accuracy (MPA) is also an important evaluation metric. Comparing the MPA of the four methods, we observe that both CNN-Visual Transformer and GCN have slightly lower MPA, but the difference is not substantial. Based on the comparison of the PA, OSCPA, NOSCPA, and MPA metrics, we find that the model proposed in this study has an overall oil spill detection capability that is roughly on par with that of the other models, with slight differences in some details. However, it also has significant advantages. The oil spill detection capabilities of the four models are quite similar, but there is a significant difference in their detection speed. The MT metric in the table shows that the method proposed in this study has a clear advantage, with an average detection time of only 696 ms per sample compared to the other three comparative methods.

**Figure 9:**
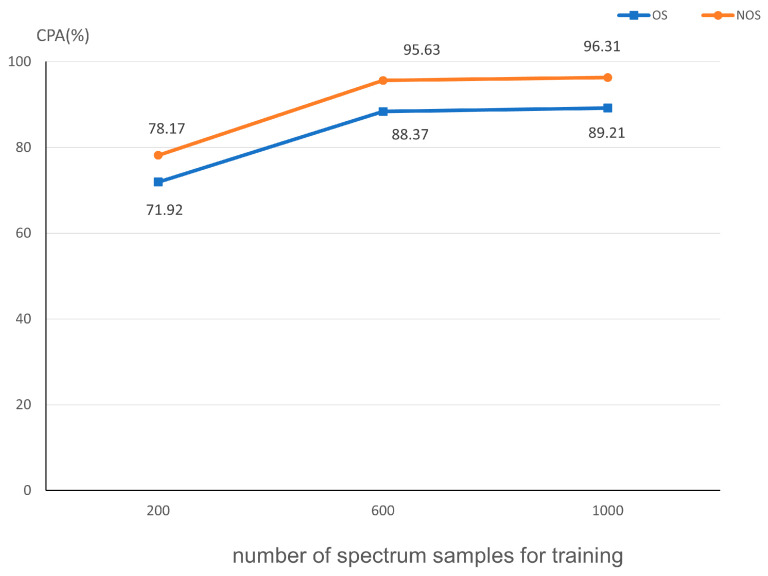
Class Pixel Accuracy under different numbers of spectrum samples for training.

To further verify the robustness of the method proposed in this study, 600 and 200 randomly selected pixel spectral samples were taken from the collected 1000 samples, and the models were retrained with these different training sample quantities. The results of testing the models retrained with three different sample sizes are shown in Figure 9.

Reducing the numbers of spectrum samples for training does lead to a decline in the performance of the method to some extent. When the numbers of spectrum samples for training are reduced to 600, the oil spill area detection performance of this method is almost unaffected, maintaining Class Pixel Accuracy at 95.63% and 88.37%. However, when the numbers of spectrum samples are further reduced to 200, there is a noticeable decline in performance, but it still maintains a Class Pixel Accuracy of over 70%. This indicates that the model proposed in this study retains some oil spill detection capability even when facing a shortage of training datasets.

## 5. Discussion

This study is based on hyperspectral image samples created in the laboratory and establishes and validates an oil spill area detection method that combines CNN and DBSCAN algorithms. However, this study has certain limitations due to constraints in the experimental environment. In a real marine environment, the scale and complexity of waves cannot be fully simulated in the laboratory, leading to unpredictable effects on hyperspectral images and detection methods. Additionally, factors such as lighting, weather conditions, and atmospheric interference can impact hyperspectral data, causing variations in the spectral characteristics of the same substance and affecting the detection performance of the method. To address these limitations, in subsequent research, we plan to move beyond the laboratory environment and collect hyperspectral images in real marine environments under different lighting conditions and weather. By enriching our dataset with images from diverse environments, the neural network model can learn deeper features, mitigating the limitations mentioned above.

During the model evaluation, we established three alternative models for comparison with the method proposed in this paper. In terms of detection accuracy, the method proposed in this study is comparable to the other three models, but it exhibits faster detection speed than the other three models. In real-time detection scenarios, only models with fast detection speed can meet the requirements for real-time detection. During maritime oil spill operations, discovering oil spill areas requires efficient and accurate detection methods. The rapid spread of pollutants in marine environments makes the real-time detection of oil spill areas crucial. If the detection speed is slow, it can increase the difficulty of oil spill response operations. Therefore, the real-time detection of oil spill areas holds significant importance. Vessels conducting operations at sea often struggle to accommodate high-powered computers. Therefore, if the model’s computational efficiency is low, data need to be transmitted to a processing center, making it challenging to meet the requirements of real-time detection. Models with lower complexity can run on smaller computers with lower computational power, allowing for direct detection. The method proposed in this study has an advantage over the other models in this regard, providing a solution for real-time offshore oil spill detection.

## 6. Conclusions

The transportation of oil by sea and offshore oil extraction, among other human activities, inevitably increase the risk of oil spills into the ocean. The timely and precise monitoring of offshore oil spill areas is crucial for responding to unexpected oil spill incidents and managing already leaked oil. Hyperspectral sensing devices are widely used in marine oil spill monitoring, but current methods face challenges in quickly and efficiently detecting these spills. This study proposes an oil spill area detection method based on the CNN model and DBSCAN algorithm. The method addresses the current issue of models not meeting real-time detection requirements, providing insights for research in this area. We simulated a maritime oil spill environment in the laboratory; created a dataset for validating oil spill detection methods using equipment such as hyperspectral cameras; and then compared three oil spill detection models, DRSNet, CNN-Visual Transformer, and GCN, with the method proposed in this study.

Based on the analysis of the experimental results, this study draws the following conclusions:

(1) Compared to DRSNet, CNN-Visual Transformer, and GCN, the model proposed in this study shows a similar level of accuracy in oil spill detection. However, it has a clear advantage in terms of detection speed when compared to the other three methods.
(2) The proposed model demonstrates high detection accuracy, even with a small number of training samples, which highlights its robustness.
(3) It outperforms the other three models in the detection of smaller oil spill areas.

The conclusions indicate that combining CNN with the DBSCAN algorithm can achieve the high-precision detection of oil spill areas, and its detection speed is faster than that of other models. This approach addresses the challenge of slow detection speeds in other models, meeting the requirements for real-time detection. It provides a solution for the real-time detection of oil spill areas at sea and can assist in remediation efforts. This has a positive impact on reducing environmental damage to the marine ecosystem.

## Figures and Tables

**Table 1:** The partitioning of hyperspectral image samples.

Experimental Conditions of the Simulation	Number of Hyperspectral Images			
	Training Set	Validation Set	Test Set	Total
No oil spill and no wave	20	15	15	50
No oil spill and waves	20	15	15	50
oil spill and no wave	142	40	20	202
oil spill and waves	142	40	20	202
**Total**	324	110	70	504

**Table 2:** Structural parameters of CNN.

Layer	Convolution Kernel	Step	Size	Activation Function
Conv1D	1 × 4	1	1 × 88	Relu
Conv1D	1 × 4	1	1 × 87	Relu
MaxPooling		2	1 × 44	
Conv1D	1 × 4	1	1 × 43	Relu
Conv1D	1 × 4	1	1 × 42	Relu
MaxPooling		2	1 × 22	
Flatten				
Dense				Relu
Dense				Relu
Dense				Relu
Dense				Softmax

**Table 3:** The confusion matrix for oil spill areas and non-oil spill areas.

		**Predict**	
		**Oil Spill**	**Non-Oil Spill**
**Actual**	**Oil Spill**	*TP*	*FN*
	**Non-Oil Spill**	*FP*	*TN*

**Table 4:** Performance metrics for the four methods.

Method	PA (%)	OSCPA (%)	NOSCPA (%)	MPA (%)	MT (ms)
CNN-DBSCAN	90.69	89.21	96.31	92.12	696
DRSNet	91.31	95.11	89.97	92.84	2386
CNN-ViT	91.93	93.09	90.81	91.57	1893
GCN	90.74	90.96	92.59	91.54	1509

## Data Availability

The core code and dataset for the methods in the article have been published at the following address: https://github.com/happyAzhan/oilspillDetection/, accessed on 25 November 2023.
